# SPPB reference values and performance in assessing sarcopenia in community-dwelling Singaporeans – Yishun study

**DOI:** 10.1186/s12877-021-02147-4

**Published:** 2021-03-30

**Authors:** Shuen Yee Lee, Pei Ling Choo, Benedict Wei Jun Pang, Lay Khoon Lau, Khalid Abdul Jabbar, Wei Ting Seah, Kenneth Kexun Chen, Tze Pin Ng, Shiou-Liang Wee

**Affiliations:** 1 Faculty of Health and Social Sciences, Singapore Institute of Technology, Singapore, Singapore; 2 School of Health and Life Sciences, Glasgow Caledonian University, Glasgow, UK; 3 Geriatric Education and Research Institute (GERI), Singapore, Singapore; 4 Department of Psychological Medicine, National University of Singapore, Singapore, Singapore

**Keywords:** Short physical performance battery, Older adults, Gait speed, Cut-off, Sit-to-stand, SPPB subtest

## Abstract

**Background:**

The Short Physical Performance Battery (SPPB) is an established test of physical performance. We provide reference values for SPPB and determine SPPB performance and cut-offs in assessing sarcopenia for Asian community-dwelling older adults.

**Methods:**

Five hundred thirty-eight (57.8% women) community-dwelling adults aged 21–90 years were recruited. SPPB and its subtest scores and timings (8 ft. gait speed (GS), five-times repeated chair sit-to-stand (STS) and balance) were determined. Appendicular lean mass divided by height-squared, muscle strength (handgrip) and physical performance (6 m GS, STS and SPPB) were assessed to define sarcopenia for various Asian criteria. Area under the ROC curve (AUC) was used to assess performance of SPPB and subtests in discriminating sarcopenia in adults aged ≥60 years. Optimal SPPB and GS subtest cut-offs for each sarcopenia criterion were determined by maximizing sensitivity and specificity.

**Results:**

The mean SPPB score was 11.6(SD 1.1) in men and 11.5(SD1.2) in women. Majority of participants(≥50%) aged 21–80 years achieved the maximum SPPB score. SPPB total and subtest scores generally decreased with age (all *p* < 0.001), but did not differ between sex. Among older adults (≥60 years), SPPB and GS subtest had varied performance in assessing sarcopenia (AUC 0.54–0.64 and 0.51–0.72, respectively), and moderate-to-excellent performance in assessing severe sarcopenia (AUC 0.69–0.98 and 0.75–0.95, respectively), depending on sarcopenia definitions. The optimal cut-offs for discriminating sarcopenia in both sexes were SPPB ≤11points and GS subtest ≤1.0 m/s. The most common optimal cut-offs for discriminating severe sarcopenia according to various definitions were SPPB ≤11points in both sexes, and GS ≤0.9 m/s in men and ≤ 1.0 m/s in women.

**Conclusions:**

Population-specific normative SPPB values are important for use in diagnostic criteria and to interpret results of studies evaluating and establishing appropriate treatment goals. Performance on the SPPB should be reported in terms of the total sum score and registered time to complete the repeated-chair STS and 8-ft walk tests. The performance of GS subtest was comparable to SPPB and could be a useful, simple and accessible screening tool for discriminating severe sarcopenia in community-dwelling older adults.

**Supplementary Information:**

The online version contains supplementary material available at 10.1186/s12877-021-02147-4.

## Background

Physical function is an important marker for health especially in older adults. Poorer physical function is associated with poorer life quality and adverse health outcomes including cognitive impairment, institutionalization and mortality [1]. Given the huge burden associated with age-related functional decline, early detection and intervention are important in mitigating poor physical function in community-dwelling adults, so as to delay functional disability and dependence [2].

The short physical performance battery (SPPB) is a valid and reliable measure of physical function in community and clinical settings [3–6]. SPPB consists of three timed components (balance, five-times repeated chair sit-to-stand (STS) and usual-pace gait speed (GS)) which measure balance, lower body muscle strength and mobility [7]. Lower SPPB scores have been shown to predict lower quality of life, loss in mobility, disability and mortality [6]. Thus, SPPB could be useful for early stratification of community-dwelling individuals at risk of functional disability, as it requires minimal time, expertise and equipment to administer [8].

SPPB has also been used as a criterion of physical performance to define sarcopenia, which is the progressive loss of muscle mass, strength and function with age [9]. Sarcopenia is associated with adverse health outcomes, such as frailty, hospitalization and mortality [9]. Several definitions and cut-offs exist for the diagnosis of sarcopenia, including the European Working Group on Sarcopenia in Older People (EWGSOP) and Asian Working Group on Sarcopenia (AWGS) [10–12]. Recently, the AWGS proposed new cut-offs for sarcopenia (AWGS19) [11], resulting in variations in sarcopenia prevalence observed, ranging from 13 to 36% in men and 12–47% in women [13]. These different definitions and cut-offs for sarcopenia could affect diagnosis and management of sarcopenia in at-risk older adults. Additionally, the recommended diagnosis of sarcopenia requires costly equipment and trained individuals to administer, which might not be ideal for population-level screening of sarcopenia. Due to the increasing prevalence of sarcopenia with an ageing population, simple markers to assess sarcopenia (e.g., SPPB) can be useful towards wider detection and management of the condition.

Since genetic, lifestyle and socioeconomic factors could affect physical performance, population-specific reference values are needed to provide meaningful interpretation and comparisons of physical function [14–16]. Therefore, the aim of this study was to provide sex- and age-specific reference values for SPPB in community-dwelling Singaporean adults aged ≥21 years. This study also determined and compared the optimal cut-offs and performance of SPPB and individual subtests (8 ft. GS and STS) in assessing sarcopenia and severe sarcopenia according to each AWGS19 criteria for physical performance, including 6 m GS, STS time and SPPB score.

## Methods

### Participants

Participants were recruited from the Yishun Study [13]. Briefly, community-dwelling adults aged 21 years and above who were independent in performing activities of daily living, had < 5 poorly-controlled comorbidities, and no neuromuscular or cognitive disorders were recruited. Random sampling methodology was used to obtain a representative sample of approximately 300 male and 300 female participants, filling quotas of 20–40 participants in each sex and age group (i.e., 10-year age groups between 21 and 60 years old and 5-year age groups after 60 years). Ethics approval was obtained from the National Healthcare Group DSRB (2017/00212), in accordance with the relevant guidelines and regulations by the Declaration of Helsinki and the ethical principles in the Belmont Report. All participants gave written informed consent to participate in the study.

### Assessments

Body weight and height were measured using an electronic scale and stadiometer respectively (SECA, Hamburg, Germany). Body mass index was calculated as body weight (kg) divided by height (m) squared. Participants declared their education level and medical conditions and comorbidities.

Handgrip strength was measured using the Jamar Plus+ Digital Hand Dynamometer (Patterson Medical, Cedarburg, WI). Participants were seated with arms in 90-degree abduction. The higher reading of two alternating trials per arm with 30s rest intervals was reported.

Usual GS was assessed using a 6 m GAITRite Walkway (CIR Systems Inc., Sparta, NJ) with 2 m lead in and out phase. Participants performed three trials and the average timing was recorded.

Body composition was determined using dual x-ray absorptiometry (Discovery WI, Hologic Inc., Marlborough, MA). Appendicular lean mass (ALM) was defined as the sum of lean mass in the upper and lower limbs. ALM corrected for height (ALM/h^2^) was calculated as ALM (kg) divided by height (m) squared.

### SPPB

The SPPB assesses lower limb function, including balance, strength and mobility. SPPB consists of 3 subtests (i.e., balance, GS and STS time) [7, 17]. The balance subtest composed of 3 parts with progressive difficulty, including unaided feet-together stand, semi-tandem stand and full-tandem stand. Participants were timed until they moved or 10s elapsed time. GS was assessed by participants walking 8 ft. at their usual pace, with a moving start [7, 17]. The average timing was recorded over two trials. To assess STS time, participants folded their arms across their chest and performed five chair stands as quickly as possible. Each of the 3 subtests was scored from 0 to 4 and the total score was the sum of 3 subtests, ranging from 0 to 12. Higher SPPB scores indicated better physical function [7].

### Sarcopenia definitions

Sarcopenia was classified according to AWGS19 definitions in participants aged ≥60 years [11]. Low muscle mass was defined as ALM/h^2^ < 7.0 and < 5.4 kg/m^2^, and low muscle strength as handgrip strength < 28 and < 18 kg, in men and women respectively. Poor physical performance was defined as GS (distance of 6 m) < 1.0 m/s, STS timing of ≥12 s or SPPB total score of ≤9. Participants were classified as sarcopenic according to 3 different AWGS19 physical performance criteria, based on cut-off fulfilment for low muscle mass (ALM/h^2^), and low muscle strength (handgrip) or poor physical performance (low GS, STS or SPPB). Participants with low muscle mass, strength and poor physical performance were classified as having severe sarcopenia.

### Statistical analysis

All statistical analyses were performed using R version 3.6.2 (R Foundation for statistical computing, Vienna, Austria). Numerical variables are presented as mean (standard deviation, SD) in text and figures unless otherwise stated. Participant characteristics were analyzed using two-way ANOVA to assess potential differences between sex and age groups. Sex differences in SPPB subtests within each age group were evaluated using T-test. The performance of SPPB and individual subtests (STS and GS) in assessing sarcopenia and severe sarcopenia among a subset of older adults aged ≥60 years were determined by calculating the area under the receiver operating characteristics curve (AUC), for each AWGS19 sarcopenia definition in men and women. AUC of 0.7–0.8 and > 0.8 were considered acceptable and excellent respectively [18]. For each AWGS19 sarcopenia physical performance criterion, optimal cut-offs for SPPB score and GS subtest were determined by the Youden index [19]. The sensitivity and specificity for each optimal cut-off value and corresponding 95% confidence intervals were reported. Age- and sex-specific smoothed centile curves for GS and STS were generated using standard analytical Lambda Mu Sigma (LMS) method with LMS ChartMakerPro v2.54 (The Institute of Health, London, United Kingdom) [4, 20–22]. A value of *p* < 0.05 was considered statistically significant.

## Results

### Participant characteristics

A total of 538 participants (57.8% women) with a mean age of 59 years (SD 19 years, range 21–90 years) were included in the analysis. Participant demographics such as age, education level and anthropometric data are summarised in Table 1. Regardless of sex, 6 m GS generally decreased with age (Table 1, *p* < 0.001). Handgrip strength and ALM/h^2^ decreased with age and were 53 and 27% higher on average in males than in females, respectively (Table 1, all *p* < 0.001).

**Table 1:** Mean (SD) Participant characteristics according to age group and sex

Age Group	21–30	31–40	41–50	51–60	61–65	66–70	71–75	76–80	> 80	All ages
**Men**										
n	28	26	20	22	29	24	29	26	23	227
Age (years)	25 (3)	36 (3)	46 (3)	57 (2)	63 (1)	68 (1)	73 (2)	78 (1)	84 (2)	59 (19)
Education, n (%)										
*< =Primary*	1 (3.5)	0 (0.0)	2 (10.0)	2 (9.0)	10 (34.4)	6 (25.0)	12 (41.3)	15 (57.6)	15 (65.2)	63 (27.7)
*Secondary*	2 (7.1)	3 (11.5)	4 (20.0)	14 (63.6)	13 (44.8)	12 (50.0)	14 (48.2)	6 (23.0)	5 (21.7)	73 (32.1)
*Tertiary*	13 (46.4)	11 (42.3)	3 (15.0)	1 (4.5)	6 (20.6)	5 (20.8)	2 (6.8)	2 (7.6)	1 (4.3)	44 (19.3)
*> =Degree*	6 (21.4)	10 (38.4)	7 (35.0)	4 (18.1)	0 (0.0)	0 (0.0)	0 (0.0)	3 (11.5)	2 (8.6)	32 (14.0)
*Other qualification*	6 (21.4)	2 (7.6)	4 (20.0)	1 (4.5)	0 (0.0)	1 (4.1)	1 (3.4)	0 (0.0)	0 (0.0)	15 (6.6)
Height (m)	1.73 (0.07)	1.70 (0.05)	1.68 (0.06)	1.69 (0.07)	1.66 (0.06)	1.65 (0.05)	1.65 (0.06)	1.63 (0.07)	1.62 (0.07)	1.67 (0.07)
Weight (kg)	80.4 (22.4)	81.2 (20)	76.8 (13.4)	73.5 (10.9)	66.2 (8)	65.9 (10.9)	65.4 (8.6)	63 (10.2)	61.6 (11.4)	70.3 (15.4)
BMI (kg/m^2^)	27.1 (8.2)	28.0 (6.7)	27.2 (3.8)	25.6 (3.2)	24.0 (2.9)	24.0 (3.4)	24.2 (3.2)	23.7 (3.0)	23.5 (4.1)	25.2 (4.9)
Gait Speed 6 m (m/s)	1.14 (0.15)	1.12 (0.19)	1.14 (0.16)	1.14 (0.17)	1.12 (0.19)	1.11 (0.17)	0.99 (0.15)	0.95 (0.21)	0.83 (0.21)	1.06 (0.20)
ALM/h^2^	7.9 (1.4)	8.2 (1.3)	7.7 (1.1)	7.7 (1.1)	6.7 (0.7)	6.6 (0.7)	6.5 (0.7)	6.4 (0.7)	6.2 (1.0)	7.1 (1.2)
Hand Grip (kg)	42.3 (8.1)	44.6 (7.4)	42.1 (6.5)	40.0 (6.7)	35.5 (5.9)	32.9 (5.9)	29.0 (7.0)	28.3 (4.8)	24.4 (7.4)	35.3 (9.4)
Total SPPB Score	12.0 (0.2)	12.0 (0.2)	12.0 (0.2)	11.9 (0.3)	11.8 (0.6)	11.9 (0.3)	11.3 (1.1)	11.2 (1.0)	10.2 (2.3)	11.6 (1.1)
Max SPPB Score (%)	96	96	95	91	90	88	62	50	39	78
SPPB Subtest score										
*Balance*	4.00 (0.00)	4.00 (0.00)	4.00 (0.00)	4.00 (0.00)	3.93 (0.37)	3.92 (0.28)	3.90 (0.41)	3.81 (0.49)	3.48 (0.85)	3.89 (0.41)
*5x Sit-to-stand*	4.00 (0.00)	4.00 (0.00)	3.95 (0.22)	3.91 (0.29)	3.90 (0.31)	3.96 (0.20)	3.48 (0.83)	3.58 (0.58)	3.30 (1.02)	3.78 (0.56)
*Gait Speed 8 ft (m/s)*	3.96 (0.19)	3.96 (0.20)	4.00 (0.00)	4.00 (0.00)	4.00 (0.00)	4.00 (0.00)	3.97 (0.19)	3.77 (0.59)	3.43 (0.79)	3.90 (0.38)
**Women**										
n	32	35	39	37	31	35	31	34	37	311
Age (years)	25 (3)	36 (3)	46 (3)	55 (3)	63 (1)	68 (1)	72 (2)	78 (1)	83 (2)	58 (19)
Education, n (%)										
*< =Primary*	0 (0.0)	0 (0.0)	3 (7.6)	9 (24.3)	11 (35.4)	19 (54.2)	17 (54.8)	23 (67.6)	29 (78.3)	111 (35.6)
*Secondary*	0 (0.0)	8 (22.8)	23 (58.9)	15 (40.5)	15 (48.3)	10 (28.5)	11 (35.4)	7 (20.5)	4 (10.8)	93 (29.9)
*Tertiary*	13 (40.6)	7 (20.0)	4 (10.2)	6 (16.2)	2 (6.4)	3 (8.5)	1 (3.2)	2 (5.8)	2 (5.4)	40 (12.8)
*> =Degree*	17 (53.1)	14 (40.0)	6 (15.3)	5 (13.5)	3 (9.6)	2 (5.7)	1 (3.2)	2 (5.8)	1 (2.7)	51 (16.3)
*Other qualification*	2 (6.2)	6 (17.1)	3 (7.6)	2 (5.4)	0 (0.0)	1 (2.8)	1 (3.2)	0 (0.0)	1 (2.7)	16 (5.1)
Height (m)	1.60 (0.05)	1.59 (0.05)	1.57 (0.07)	1.57 (0.06)	1.55 (0.05)	1.54 (0.05)	1.53 (0.05)	1.52 (0.05)	1.48 (0.04)	1.55 (0.06)
Weight (kg)	57.7 (11.7)	61.6 (12.3)	63.4 (11.7)	63.1 (14.1)	58.8 (8.7)	59.3 (7.6)	54.0 (8.4)	57.5 (8.2)	52.8 (8.6)	58.8 (10.9)
BMI (kg/m^2^)	22.5 (4.5)	24.5 (4.7)	25.7 (4.3)	25.6 (5.5)	24.4 (3.6)	25.0 (3.0)	23.0 (3.6)	25.0 (3.5)	24.2 (4.0)	24.5 (4.2)
Gait Speed 6 m (m/s)	1.14 (0.18)	1.14 (0.13)	1.18 (0.20)	1.14 (0.16)	1.09 (0.14)	1.05 (0.18)	1.01 (0.14)	0.90 (0.17)	0.83 (0.16)	1.05 (0.20)
ALM/h^2^	5.4 (0.9)	5.8 (1.0)	6.0 (1.0)	6.0 (1.3)	5.5 (0.7)	5.6 (0.7)	5.2 (0.7)	5.4 (0.7)	5.2 (0.7)	5.6 (0.9)
Hand Grip (kg)	25.7 (4.7)	26.2 (4.6)	27.7 (5.3)	23.7 (4.1)	23.1 (3.7)	22.8 (4.5)	21.0 (4.1)	19.6 (4.1)	17.9 (3.4)	23.1 (5.3)
Total SPPB Score	12.0 (0.2)	11.9 (0.5)	12.0 (0.0)	11.8 (0.4)	11.6 (1.2)	11.6 (1.2)	11.6 (0.8)	11.0 (1.4)	10.4 (2.1)	11.5 (1.2)
Max SPPB Score (%)	97	94	100	84	81	83	77	50	32	77
SPPB Subtest score										
*Balance*	4.00 (0.00)	4.00 (0.00)	4.00 (0.00)	4.00 (0.00)	3.84 (0.73)	3.97 (0.17)	3.87 (0.34)	3.79 (0.59)	3.43 (0.96)	3.88 (0.49)
*5x Sit-to-stand*	4.00 (0.00)	3.89 (0.53)	4.00 (0.00)	3.89 (0.31)	3.81 (0.54)	3.71 (0.71)	3.81 (0.54)	3.56 (0.82)	3.30 (0.97)	3.77 (0.62)
*Gait Speed 8 ft (m/s)*	3.97 (0.18)	4.00 (0.00)	4.00 (0.00)	3.95 (0.23)	3.94 (0.36)	3.91 (0.37)	3.97 (0.18)	3.65 (0.65)	3.62 (0.72)	3.89 (0.41)

*SPPB total score of maximum 12 points, which consists of 3 subtest scores of maximum 4 points each (Balance, 5x sit-to-stand time, Gait Speed (8-ft))*

### SPPB

The mean total SPPB score was approximately 12 points (maximum score) until ages 66–70 in men and 71–75 in women (Table 1). Apart from those aged > 80 years, ≥50% of men and women in all age groups achieved the maximum SPPB score (Table 1).

SPPB total and subtest scores generally decreased with age (all *p* < 0.001) but did not differ between sex (SPPB *p* = 0.706;STS *p* = 0.798;GS *p* = 0.737;balance *p* = 0.710) (Table 1). Individual SPPB subtest scores were also approximately 4 points (maximum score) until age 76–80 in both men and women (Table 1). The age-related decline in SPPB GS was apparent above ages ~ 70 and ~ 75 years in men and women respectively, with men showing a steeper decline in GS than women (Fig. S1A). SPPB GS was generally 5% higher in men than women (Table 2, *p* = 0.017). Specifically, SPPB GS was 12% higher in men than in women aged 51–60 years (Table 2, *p* = 0.013). Compared with men, women had poorer performance in STS time until age ~ 70 years, while men performed worse after ~ 70 years (Fig. S1B). STS time was 15% lower in men than in women aged 61–65 years (Table 2, *p* = 0.029).

**Table 2:** Mean (SD) of SPPB subtests (5 times repeated chair sit-to-stand time and gait speed) between men and women across different age groups

	Men	Women	***p*** value
**Five times sit-to-stand, s**			
21–30	7.1 (1.1)	7.5 (1.1)	0.122
31–40	8.0 (1.2)	8.9 (2.8)	0.086
41–50	7.7 (1.5)	8.0 (1.5)	0.502
51–60	8.4 (1.9)	9.0 (1.8)	0.237
61–65	8.3 (2.0)	9.6 (2.2)	0.029*
66–70	8.9 (1.5)	9.8 (2.8)	0.114
71–75	10.7 (4.0)	9.3 (2.0)	0.100
76–80	10.4 (2.1)	10.3 (2.7)	0.927
> 80	11.4 (3.1)	10.9 (3.0)	0.523
All ages	9.0 (2.6)	9.3 (2.5)	0.255
**Gait speed (8 ft), m/s**			
21–30	1.27 (0.21)	1.18 (0.16)	0.083
31–40	1.25 (0.22)	1.19 (0.16)	0.263
41–50	1.22 (0.12)	1.24 (0.21)	0.732
51–60	1.28 (0.20)	1.14 (0.20)	0.013*
61–65	1.18 (0.21)	1.13 (0.17)	0.276
66–70	1.19 (0.17)	1.10 (0.17)	0.066
71–75	1.06 (0.17)	1.11 (0.16)	0.227
76–80	1.01 (0.31)	0.92 (0.19)	0.241
> 80	0.89 (0.28)	0.88 (0.20)	0.893
All ages	1.15 (0.25)	1.10 (0.21)	0.017*

*p < 0.05

Sex- and age-specific percentile reference values for SPPB subtests GS and STS are presented in Supplementary Table S1.

### Characteristics of participants ≥60 years with sarcopenia and severe sarcopenia

A total of 303 participants (55% women) aged ≥60 years were analysed separately to determine the ability of SPPB in assessing sarcopenia in our population. Baseline characteristics of participants with sarcopenia and severe sarcopenia, classified according to 3 different AWGS19 physical performance criteria, are presented in Table 3.

**Table 3:** Mean (SD) characteristics of sarcopenic and severe sarcopenic men and women aged ≥60 years according to each AWGS19 physical performance definitions for sarcopenia, including 6 m Gait Speed (GS), 5x repeated-chair Sit-to-Stand (STS) and Short Physical Performance Battery (SPPB)

	Sarcopenia			Severe Sarcopenia		
	AWGS19 GS	AWGS19 STS	AWGS19 SPPB	AWGS19 GS	AWGS19 STS	AWGS19 SPPB
**Males**						
*n*	29	34	33	27	11	8
Age (years)	74 (7)	76 (7)	76 (7)	78 (7)	78 (7)	80 (8)
Height (m)	1.62 (0.05)	1.62 (0.07)	1.61 (0.07)	1.62 (0.08)	1.65 (0.07)	1.63 (0.10)
Weight (kg)	60.2 (6.3)	59.5 (7.7)	59.0 (6.5)	61.1 (8.2)	64.1 (8.5)	64.1 (11.2)
BMI	22.9 (2.2)	22.7 (2.2)	22.6 (2.1)	23.4 (2.9)	23.8 (3.7)	24.2 (4.1)
ALM/h^2^	6.1 (0.5)	5.9 (0.5)	5.9 (0.5)	6.0 (0.5)	6.0 (0.5)	5.9 (0.6)
Hand Grip (kg)	28.7 (5.2)	25.0 (6.8)	23.2 (3.9)	21.5 (4.7)	20.8 (4.1)	20.0 (6.3)
Gait Speed 6 m (m/s)	0.95 (0.15)	0.93 (0.20)	0.90 (0.17)	0.83 (0.19)	0.75 (0.15)	0.66 (0.14)
SPPB score	11.4 (1.5)	11.0 (1.6)	11.1 (1.5)	10.3 (1.9)	9.0 (2.0)	8.0 (1.7)
**Females**						
*n*	42	28	36	24	11	2
Age (years)	75 (7)	76 (6)	78 (6)	80 (6)	81 (4)	84 (0)
Height (m)	1.53 (0.04)	1.51 (0.06)	1.51 (0.06)	1.50 (0.06)	1.52 (0.04)	1.54 (0.04)
Weight (kg)	52.6 (6.6)	51.6 (7.5)	51.7 (7.8)	52.3 (8.0)	53.4 (8.0)	50.3 (6.4)
BMI	22.5 (2.7)	22.6 (2.9)	22.7 (3.1)	23.2 (2.8)	23.1 (3.1)	21.5 (3.7)
ALM/h^2^	4.9 (0.3)	4.8 (0.4)	4.8 (0.4)	4.8 (0.3)	4.8 (0.4)	4.7 (0.7)
Hand Grip (kg)	20.5 (4.2)	16.5 (3.2)	16.1 (2.5)	15.5 (1.9)	15.3 (1.6)	12.8 (0.1)
Gait Speed 6 m (m/s)	0.93 (0.15)	0.90 (0.16)	0.89 (0.17)	0.87 (0.15)	0.86 (0.13)	0.65 (0.01)
SPPB score	11.3 (1.7)	11.0 (1.5)	10.9 (1.4)	10.7 (1.5)	9.7 (1.7)	6.5 (0.7)

Sarcopenia prevalence ranged from 21–25% in males and 17–25% in females, with the highest prevalence evident in STS criteria in men and GS criteria in women (Table 4). Prevalence for sarcopenia was lowest in GS criteria in men and STS criteria in women. Prevalence of severe sarcopenia in the community ranged from 6 to 20% in men and 1–14% in women, with the highest prevalence evident in GS criteria and lowest prevalence in SPPB criteria in both sexes (Table 4).

**Table 4:** Optimal cut-off values and performance of SPPB total score and SPPB gait speed (8 ft) subtest in assessing sarcopenia and severe sarcopenia according to each AWGS19 physical performance criteria, including 6 m Gait Speed (GS), 5x repeated-chair Sit-to-Stand (STS) and Short Physical Performance Battery (SPPB), in men and women aged ≥60 years

	Prevalence (%)	SPPB (Total score)			SPPB subtest: Gait Speed (8 ft) (m/s)		
		Cut-off	Sensitivity (95% CI)	Specificity (95% CI)	Cut-off	Sensitivity (95% CI)	Specificity (95% CI)
**Males (total** ***n*** **= 136)**							
**Sarcopenia**							
AWGS19 GS	21.3	6	7% (1, 23)	99% (95, 100)	1.2	86% (68, 96)	33% (24, 42)
AWGS19 STS	25.0	11	44% (27, 62)	71% (61, 79)	1.0	68% (49, 83)	72% (62, 80)
AWGS19 SPPB	24.3	11	42% (25, 61)	70% (60, 79)	1.0	70% (51, 84)	72% (62, 80)
**Severe Sarcopenia**							
AWGS19 GS	19.9	11	67% (46, 83)	75% (66, 83)	0.9	70% (50, 86)	91% (84, 96)
AWGS19 STS	8.1	11	100% (72, 100)	73% (64, 80)	0.9	82% (48, 98)	84% (76, 90)
AWGS19 SPPB	5.9	9	100% (63, 100)	97% (92, 99)	0.9	100% (63, 100)	84% (76, 90)
**Females (total** ***n*** **= 167)**							
**Sarcopenia**							
AWGS19 GS	25.1	10	19% (9, 34)	82% (74, 88)	1.1	71% (55, 84)	42% (33, 51)
AWGS19 STS	16.9	10	29% (13, 49)	84% (77, 90)	1.0	57% (37, 76)	68% (60, 76)
AWGS19 SPPB	21.6	11	58% (41, 74)	69% (61, 77)	1.0	64% (46, 79)	71% (62,79)
**Severe Sarcopenia**							
AWGS19 GS	14.4	11	71% (49, 87)	69% (61, 77)	1.0	83% (63, 95)	71% (63, 79)
AWGS19 STS	6.6	11	100% (72, 100)	68% (60, 76)	1.0	82% (48, 98)	67% (59, 74)
AWGS19 SPPB	1.2	7	100% (16, 100)	96% (91, 98)	0.7	100% (14, 100)	93% (88, 97)

### AUCs for sarcopenia and severe sarcopenia with SPPB and subtests

**Fig. 1:**
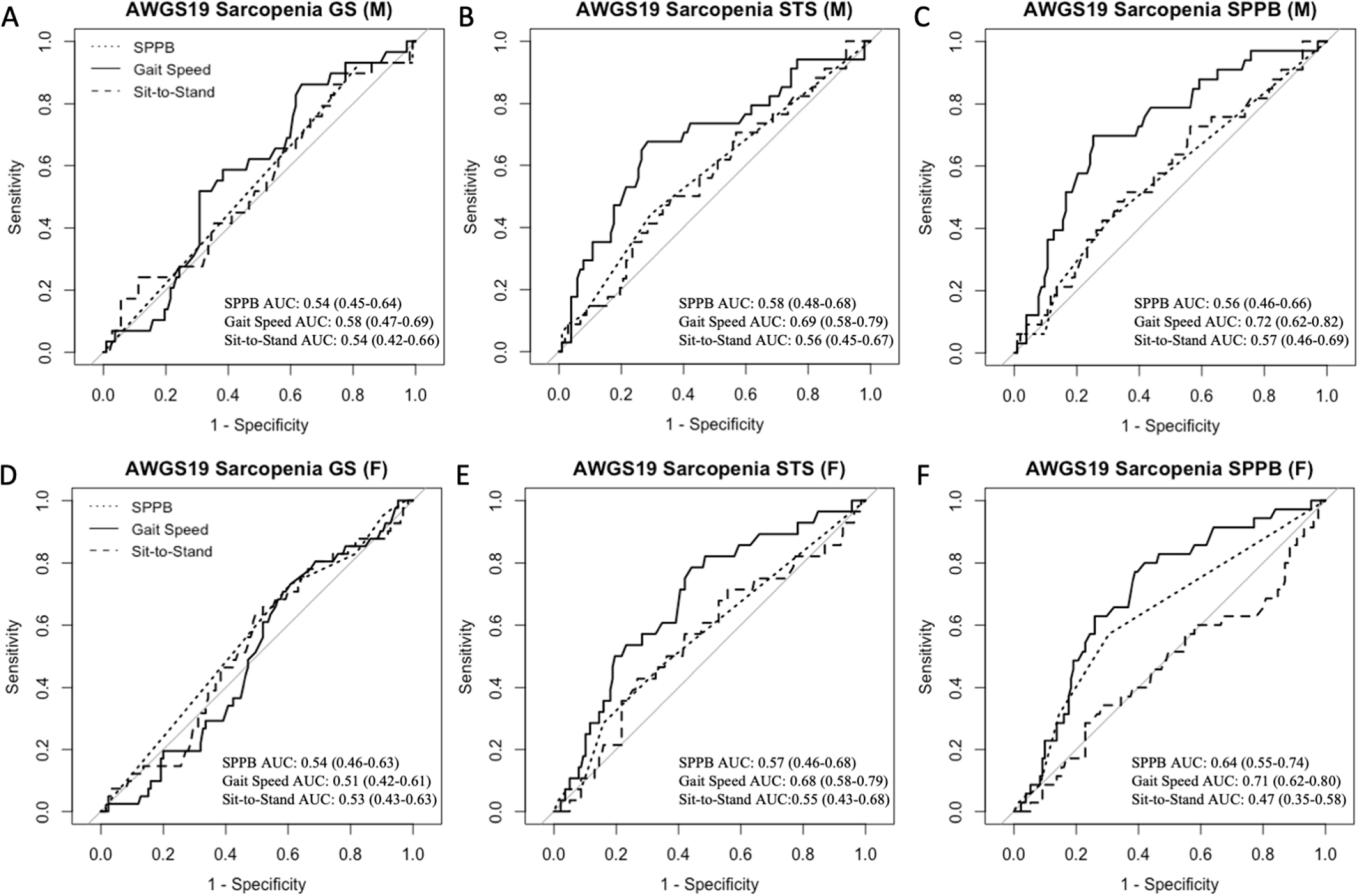
Receiver operating characteristics (ROC) curves performance for total SPPB score (dotted line), SPPB subtest for gait speed (solid line) and SPPB subtest for sit-to-stand (dashed line) in assessing sarcopenia in men (M) (**a–c**) and women (F) (**d–f**) aged ≥60, according to each AWGS19 physical performance definition for sarcopenia, including gait speed (GS) (**a,d**), sit-to-stand (STS) (**b,e**) and Short Physical Performance Battery (SPPB) (**c,f**). Respective area under the ROC curve (AUC) and 95% CI are presented

SPPB had limited ability in assessing sarcopenia according to different AWGS19 definitions, with AUC ranging from 0.54–0.58 in males (Fig. 1a–c) and 0.54–0.64 in females (Fig. 1d–f). SPPB performed the best using STS and SPPB as sarcopenia criteria in men and women respectively (Fig. 1b&f). SPPB had the worst performance for assessing sarcopenia based on GS criteria in men and women (Fig. 1a&d).

GS subtest had moderate performance in assessing sarcopenia, with AUC ranging from 0.58–0.72 in men and 0.51–0.71 in women, depending on AWGS19 criteria (Fig. 1). GS subtest performed the best for SPPB criteria (Fig. 1c&f) and worst for GS criteria (Fig. 1a&d) in both men and women. STS subtest was not useful in assessing sarcopenia, with AUC ranging from 0.54–0.57 in men and 0.47–0.55 in women, depending on definitions (Fig. 1). Hence, STS subtest was not included in the analysis for optimal cut-off values in discriminating sarcopenia.

**Fig. 2:**
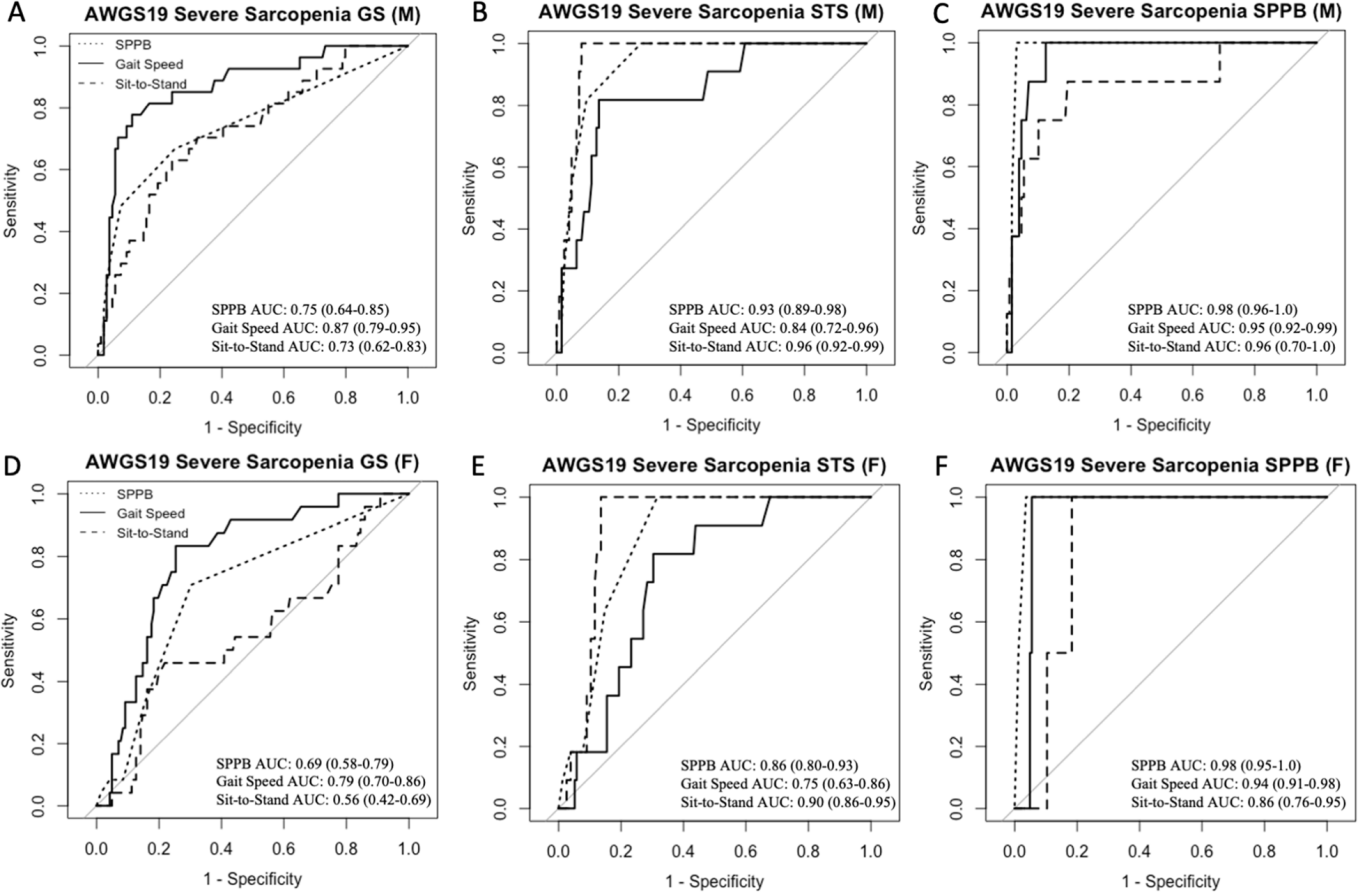
Receiver operating characteristics (ROC) curves performance for total SPPB score (dotted line), SPPB subtest for gait speed (solid line) and SPPB subtest for sit-to-stand (dashed line) in assessing severe sarcopenia in men (M) (**a–c**) and women (F) (**d–f**) aged ≥60, according to each AWGS19 physical performance definition for severe sarcopenia, including gait speed (GS) (**a,d**), sit-to-stand (STS) (**b,e**) and Short Physical Performance Battery (SPPB) (**c,f**). Respective area under the ROC curve (AUC) and 95% CI are presented

In both sexes, SPPB had excellent ability in discriminating severe sarcopenia based on AWGS19 STS and SPPB definitions (AUC 0.86–0.98) and moderate performance for AWGS19 GS criteria (AUC 0.69–0.75) (Fig. 2). GS subtest had moderate-to-excellent performance in assessing severe sarcopenia for all criteria (AUC 0.75–0.95, Fig. 2). STS subtest had excellent ability in assessing severe sarcopenia for AWGS19 STS and SPPB definitions (AUC 0.86–0.96, Fig. 2b–c&e–f) but was less useful in assessing severe sarcopenia for AWGS19 GS criteria (AUC 0.56–0.73, Fig. 2a&d).

### Performance of SPPB and GS cut-offs in assessing sarcopenia

The most commonly observed optimal SPPB cut-offs for sarcopenia was ≤11 in men (sensitivity 42–44%, specificity 70–71%) and ≤ 10 (sensitivity 19–29%, specificity 82–84%) in women. SPPB cut-off of ≤11 points had the best sensitivity (44 and 58%) and specificity (71 and 69%) for STS sarcopenia criteria in men and SPPB sarcopenia criteria in women respectively (Table 4). For severe sarcopenia, the most commonly observed optimal SPPB cut-off was ≤11 in both men and women (Table 4). In 8 men and 2 women with severe sarcopenia based on AWGS19 SPPB definition, SPPB cut-off of ≤9 and ≤ 7 in men and women respectively, gave the best sensitivity and specificity (Table 4).

The most frequent GS cut-off for sarcopenia was ≤1.0 m/s in both men (sensitivity 68–70%, specificity 72%) and women (sensitivity 57–64%, specificity 68–71%) (Table 4). The GS cut-off at ≤1.0 m/s also gave the best sensitivity and specificity for the SPPB sarcopenia criteria in men and women (Table 4).

For severe sarcopenia, the most common optimal GS cut-off was ≤0.9 m/s in men and ≤ 1.0 m/s in women (Table 4). In two women with severe sarcopenia based on SPPB criteria, GS cut-off of 0.7 m/s gave the highest sensitivity and specificity (Table 4).

## Discussion

The present study is the first to report sex-specific reference values for SPPB among community-dwelling adults aged 21–80+ years old in Singapore. Among older adults aged ≥60 years, SPPB and GS subtest had varied performance in assessing sarcopenia but moderate-to-excellent performance for discriminating severe sarcopenia. We show that SPPB cut-off score of ≤11 had optimal sensitivity and specificity for discriminating sarcopenia and severe sarcopenia in this Asian cohort of community-dwelling older adults, based on various AWGS19 definitions. The study results also suggest that regardless of sex, the GS subtest could be useful in assessing sarcopenia in our population.

In our study population, more than half of participants aged 21–80 years and over a third participants aged > 80 years achieved the maximum SPPB score of 12. This implies a ceiling effect for SPPB in our population, as > 20% of men and women across all age groups achieved the highest possible score [23]. Our findings agree with a Norwegian study which reported ceiling effects of SPPB, across age groups 40–80+ years [3]. However, such a ceiling effect of SPPB was not observed in Colombian adults aged > 80 years, with 19.8% of males and 7% of females with an SPPB score of 10–12 [4]. Across ages 40–80+ years, mean SPPB scores in men and women were similar between our study participants and Norwegian adults [3]. Compared with Colombian older adults (60–80+ years), the mean SPPB in our population was higher in both sexes (by ~ 2–3 points) [4]. These findings suggest that SPPB scores differ by population and population-specific reference values are necessary. The presence of ceiling effects in our population support the need to report specific SPPB subtest values, rather than aggregated scores, in order to better classify physical performance in community-dwelling older adults with higher functional ability. The disparity in SPPB scores between populations could be due to socio-economic, racial or ethnicity differences. For example, poverty and lower education were associated with greater likelihood of physical functioning limitation among older adults aged > 60 years [15]. In older adults aged ≥65 years, non-Hispanic blacks had poorer SPPB scores and greater mobility disability than non-Hispanic whites, implying that race and genetic factors could also affect physical function [14]. Therefore, it is important to report population-specific SPPB and individual subtest values in community-dwelling adults.

Sarcopenia is associated with functional decline, increased risk of frailty, falls and mortality [24], which contribute to huge personal, social and economic burdens [25]. In our study, the prevalence of sarcopenia and severe sarcopenia combined ranged from 30–41% in men and 23–40% in women depending on AWGS19 definition, justifying the need for markers such as SPPB, to assess sarcopenia and poor physical function in a quicker and easier manner among the wider population. The prevalence of severe sarcopenia among Caucasian older adults was 7 and 8% according to EWGSOP2 SPPB and GS criteria respectively [26]. The difference in prevalence between definitions was smaller than our study of 3 and 17% for AWGS19 SPPB and GS criteria respectively. These differences could be attributed to the different cut-offs for SPPB and GS between EWGSOP2 and AWGS19 for the diagnosis of severe sarcopenia. The varied sarcopenia prevalence by definition also highlights the importance of comparison across different physical performance criteria, for the performance of SPPB and its subtests in discriminating sarcopenia.

The present study showed that SPPB cut-point of ≤ 11 gave the optimal sensitivity (42–58%) and specificity (69–71%) for assessing sarcopenia in community-dwelling adults ≥60 years. For severe sarcopenia in men and women, SPPB cut-off of ≤11 had optimal performance for AWGS19 GS and STS definitions. Although SPPB cut-off of ≤9 in men and ≤ 7 in women gave the best sensitivity and specificity for AWGS19 SPPB definition for severe sarcopenia, these results should be interpreted with caution due to the small sample size of men (*n =* 8) and women (*n* = 2) with severe sarcopenia based on the AWGS19 SPPB criterion. The cut-off of ≤11 was higher than the recommended SPPB cut-point of ≤8–9 suggested by EWGSOP and AWGS19 SPPB criteria for sarcopenia [10, 11, 26, 27]. Other studies also reported SPPB cut-points of 7–9 being associated with higher mortality risk [28–30]. The optimal SPPB cut-point of ≤11 for discriminating severe sarcopenia in our study was also higher than an Australian study which showed an optimal SPPB cut-point of 5–8, depending on physical performance definition such as GS and SPPB, for severe sarcopenia [26]. Differences in study populations likely explain the disparity. Our study participants were community-dwelling older Asian adults with high functional ability, which differed from other studies involving Caucasians [26, 29], outpatient or hospitalised individuals who might have limited physical function [28, 30]. Furthermore, SPPB scores are commonly stratified into groups (0–3, 4–6, 7–9, 10–12), with a score of 10–12 as the reference (normal) group [7, 27, 31]. Within individuals with SPPB score 10–12, varying physical function, risk of sarcopenia and mortality plausibly exist. Compared with individuals with maximum SPPB score, individuals with score of 11 were 1.4 times more likely to develop mobility disability in a 3-year follow-up study [32]. These results suggest that a 1-point decrease in SPPB score could impact physical function [33]. Therefore, in functional community-dwelling older adults, a higher SPPB cut-off can better discriminate sarcopenia. Nonetheless, SPPB had limited performance in discriminating sarcopenia in our study, despite moderate-to-excellent performance in assessing severe sarcopenia. These results suggest that the cut-point of ≤11points might be more useful for assessing severe sarcopenia among community-dwelling older adults.

We compared the performance of individual SPPB subtests in assessing sarcopenia. Our results demonstrate that GS, but not STS subtest, generally had better performance than total SPPB score in discriminating sarcopenia, and had comparable performance with SPPB in assessing severe sarcopenia. Among our participants, GS subtest cut-off of ≤ 1.0 m/s gave optimal sensitivity (57–70%) and specificity (68–72%) in assessing sarcopenia in men and women for STS and SPPB criteria. For discriminating severe sarcopenia, optimal GS subtest cut-off was ≤1.0 m/s in women (GS and STS criteria) and ≤ 0.9 m/s in men (all AWGS19 criteria). Our findings agree with the recommended AWGS19 cut-off for GS criteria, despite a different walk distance of 6 m in AWGS19 and 8 ft. in the present study [11]. Other studies also reported a GS of < 1.0 m/s in sarcopenic older adults [34], and found greater dementia risk and poorer health outcomes in adults > 80 years with GS of < 1.0 m/s [35, 36]. However, GS cut-off recommendations varied according to sarcopenia-associated health outcomes, such as hospitalisation, falls, mortality, cognitive impairment [37]. For example, other studies including the EWGSOP recommended a GS cut-off of 0.8 m/s [10, 12, 38], due to its association with lower life expectancy [39] and disability [37]. Nonetheless, GS cut-offs are dependent on health status and demographics, supporting the need for population-specific studies investigating the diagnostic value of GS in sarcopenia. Herein, we propose that the GS subtest of SPPB might be a useful, simple and accessible tool for assessing sarcopenia in functional community-dwelling older adults.

Our study used a well-established performance-based physical function assessment and recruited randomly from the general population, suggesting a good degree of generalisability. However, the study findings cannot be generalised to people living in institutions. Future longitudinal studies should investigate the prognostic value of SPPB and its subtests in diagnosing sarcopenia and severe sarcopenia.

## Conclusions

Our study provides normative SPPB values in community-dwelling Singaporean adults aged 21–90 years, which is important due to differences in physiological and lifestyle factors between populations, and to provide comparative references with diseased states. Performance on the SPPB should be reported in terms of the total sum score and registered time to complete the repeated chair STS and 8-ft walk tests. Our results suggest that GS subtest performed better than SPPB in discriminating sarcopenia and had comparable performance with SPPB for assessing severe sarcopenia. GS subtest could be a useful, simple and accessible tool for assessing severe sarcopenia in the community.

## Supplementary Information

**Additional file 1: Supplementary Table S1.** Smooth centile scores and LMS values for SPPB subtest: Gait speed (m/s) test and Sit-to-stand time (s) according to age and sex. **Supplementary Fig. S1.** Smooth local regression and 95% confidence intervals for individual SPPB subtests: 8-ft gait speed (A) and 5-times repeated chair sit-to-stand time (B) in males (black line, triangle) and females (grey line, circle).

## Data Availability

The datasets used and/or analysed during the current study are available from the corresponding author on reasonable request.

## Abbreviations

SPPB: Short physical performance battery
STS: Sit-to-stand
EWGSOP: European Working Group on Sarcopenia in Older People
AWGS: Asian Working Group on Sarcopenia
GS: Gait speed
ALM: Appendicular lean mass
ALM/h^2^: Appendicular lean mass corrected for height
AUC: Area under the receiver operating characteristics curve
LMS: Lamda Mu Sigma
