# Supplementary material for: SPPB reference values and performance in assessing sarcopenia in community-dwelling Singaporeans – Yishun study
Source: BMC Geriatr. 2021 Mar 30;21:213. doi: 10.1186/s12877-021-02147-4 (PMC8008740; doi:10.1186/s12877-021-02147-4)
Supplement: Supplementary file 1 — Additional file 1: Supplementary Table S1. Smooth centile scores and LMS values for SPPB subtest: Gait speed (m/s) test and Sit-to-stand time (s) according to age and sex. Supplementary Fig. S1. Smooth local regression and 95% confidence intervals for individual SPPB subtests: 8-ft gait speed (A) and 5-times repeated chair sit-to-stand time (B) in males (black line, triangle) and females (grey line, circle). [file 12877_2021_2147_MOESM1_ESM.docx]

**Title**: SPPB reference values and performance in assessing sarcopenia in community-dwelling Singaporeans – Yishun Study

Shuen Yee Lee, PhD^a^, Pei Ling Choo, PhD^a,b^, Benedict Wei Jun Pang, BSc^c^, Lay Khoon Lau, PhD^c^, Khalid Abdul Jabbar, MSc^c^, Wei Ting Seah, MSc^c^, Kenneth Kexun Chen, BSc^c^, Tze Pin Ng, MD^c,d^, Shiou-Liang Wee, PhD^a,c^

^a^Faculty of Health and Social Sciences, Singapore Institute of Technology, Singapore

^b^School of Health and Life Sciences, Glasgow Caledonian University, United Kingdom

^c^Geriatric Education and Research Institute, Singapore

^d^Department of Psychological Medicine, National University of Singapore, Singapore

**Corresponding author:** Shiou-Liang Wee, Geriatric Education and Research Institute (GERI), 2 Yishun Central 2, Tower E Level 4 GERI Admin, 768024, Singapore. Phone: +65 65924606, Email: [weeshiouliang@gmail.com](mailto:weeshiouliang@gmail.com)

**Supplementary Table and Figure**

**Supplementary Table S1.** Smooth centile scores and LMS values for SPPB subtest: Gait speed (m/s) test and Sit-to-stand time (s) according to age and sex.

| **Age (years)** | **L** | **S** | **5th** | **10th** | **25th** | **50th** | **75th** | **90th** | **95th** |
| --- | --- | --- | --- | --- | --- | --- | --- | --- | --- |
| **SPPB subtest: Gait speed (m/s)** | | | | | | | | | |
| **Men** |  |  |  |  |  |  |  |  |  |
| 25 | 1.15 | 0.21 | 0.80 | 0.90 | 1.06 | 1.23 | 1.40 | 1.56 | 1.65 |
| 30 | 1.15 | 0.21 | 0.79 | 0.89 | 1.05 | 1.22 | 1.39 | 1.54 | 1.63 |
| 35 | 1.15 | 0.21 | 0.78 | 0.88 | 1.04 | 1.21 | 1.38 | 1.53 | 1.62 |
| 40 | 1.15 | 0.21 | 0.78 | 0.87 | 1.03 | 1.20 | 1.37 | 1.52 | 1.61 |
| 45 | 1.15 | 0.21 | 0.77 | 0.87 | 1.02 | 1.19 | 1.36 | 1.51 | 1.59 |
| 50 | 1.15 | 0.21 | 0.76 | 0.86 | 1.01 | 1.18 | 1.35 | 1.49 | 1.58 |
| 55 | 1.15 | 0.21 | 0.76 | 0.85 | 1.00 | 1.17 | 1.33 | 1.48 | 1.56 |
| 60 | 1.15 | 0.21 | 0.75 | 0.84 | 0.99 | 1.16 | 1.32 | 1.46 | 1.54 |
| 65 | 1.15 | 0.21 | 0.74 | 0.83 | 0.98 | 1.14 | 1.30 | 1.44 | 1.52 |
| 70 | 1.15 | 0.21 | 0.73 | 0.82 | 0.96 | 1.12 | 1.28 | 1.42 | 1.50 |
| 75 | 1.15 | 0.21 | 0.71 | 0.80 | 0.95 | 1.10 | 1.26 | 1.39 | 1.47 |
| 80 | 1.15 | 0.21 | 0.70 | 0.79 | 0.93 | 1.09 | 1.24 | 1.37 | 1.45 |
| 85 | 1.15 | 0.21 | 0.69 | 0.77 | 0.91 | 1.07 | 1.21 | 1.35 | 1.42 |
| **Women** |  |  |  |  |  |  |  |  |  |
| 25 | 1.28 | 0.19 | 0.78 | 0.87 | 1.01 | 1.15 | 1.30 | 1.42 | 1.50 |
| 30 | 1.28 | 0.19 | 0.78 | 0.86 | 1.00 | 1.15 | 1.29 | 1.42 | 1.49 |
| 35 | 1.28 | 0.19 | 0.77 | 0.86 | 1.00 | 1.14 | 1.29 | 1.41 | 1.48 |
| 40 | 1.28 | 0.19 | 0.77 | 0.85 | 0.99 | 1.14 | 1.28 | 1.40 | 1.48 |
| 45 | 1.28 | 0.19 | 0.77 | 0.85 | 0.99 | 1.13 | 1.27 | 1.40 | 1.47 |
| 50 | 1.28 | 0.19 | 0.76 | 0.84 | 0.98 | 1.13 | 1.26 | 1.39 | 1.46 |
| 55 | 1.28 | 0.19 | 0.76 | 0.84 | 0.97 | 1.12 | 1.26 | 1.38 | 1.45 |
| 60 | 1.28 | 0.19 | 0.75 | 0.83 | 0.96 | 1.11 | 1.24 | 1.36 | 1.44 |
| 65 | 1.28 | 0.19 | 0.74 | 0.82 | 0.95 | 1.10 | 1.23 | 1.35 | 1.42 |
| 70 | 1.28 | 0.19 | 0.73 | 0.81 | 0.94 | 1.08 | 1.22 | 1.34 | 1.40 |
| 75 | 1.28 | 0.19 | 0.72 | 0.80 | 0.93 | 1.07 | 1.20 | 1.32 | 1.39 |
| 80 | 1.28 | 0.19 | 0.71 | 0.79 | 0.92 | 1.06 | 1.19 | 1.30 | 1.37 |
| 85 | 1.28 | 0.19 | 0.70 | 0.78 | 0.91 | 1.04 | 1.17 | 1.28 | 1.35 |
| **SPPB subtest: Five times repeated chair sit-to-stand time (s)** | | | | | | | | | |
| **Men** |  |  |  |  |  |  |  |  |  |
| 25 | -0.54 | 0.24 | 5.23 | 5.63 | 6.40 | 7.48 | 8.85 | 10.46 | 11.64 |
| 30 | -0.54 | 0.24 | 5.34 | 5.74 | 6.53 | 7.63 | 9.03 | 10.67 | 11.87 |
| 35 | -0.54 | 0.24 | 5.44 | 5.85 | 6.66 | 7.78 | 9.21 | 10.88 | 12.11 |
| 40 | -0.54 | 0.24 | 5.55 | 5.97 | 6.79 | 7.93 | 9.39 | 11.09 | 12.34 |
| 45 | -0.54 | 0.24 | 5.66 | 6.08 | 6.92 | 8.08 | 9.57 | 11.30 | 12.58 |
| 50 | -0.54 | 0.24 | 5.77 | 6.20 | 7.06 | 8.24 | 9.76 | 11.53 | 12.83 |
| 55 | -0.54 | 0.24 | 5.88 | 6.33 | 7.20 | 8.41 | 9.95 | 11.75 | 13.08 |
| 60 | -0.54 | 0.24 | 6.00 | 6.45 | 7.34 | 8.58 | 10.15 | 11.99 | 13.35 |
| 65 | -0.54 | 0.24 | 6.12 | 6.59 | 7.50 | 8.75 | 10.36 | 12.24 | 13.63 |
| 70 | -0.54 | 0.24 | 6.25 | 6.73 | 7.66 | 8.94 | 10.59 | 12.50 | 13.92 |
| 75 | -0.54 | 0.24 | 6.39 | 6.87 | 7.82 | 9.13 | 10.81 | 12.77 | 14.22 |
| 80 | -0.54 | 0.24 | 6.53 | 7.02 | 7.99 | 9.33 | 11.05 | 13.05 | 14.53 |
| 85 | -0.54 | 0.24 | 6.67 | 7.17 | 8.16 | 9.53 | 11.29 | 13.33 | 14.84 |
| **Women** |  |  |  |  |  |  |  |  |  |
| 25 | -0.38 | 0.24 | 5.97 | 6.45 | 7.38 | 8.65 | 10.24 | 12.04 | 13.32 |
| 30 | -0.38 | 0.24 | 5.98 | 6.47 | 7.40 | 8.67 | 10.27 | 12.07 | 13.36 |
| 35 | -0.38 | 0.24 | 6.00 | 6.49 | 7.42 | 8.70 | 10.30 | 12.10 | 13.40 |
| 40 | -0.38 | 0.24 | 6.02 | 6.50 | 7.44 | 8.72 | 10.33 | 12.14 | 13.44 |
| 45 | -0.38 | 0.24 | 6.04 | 6.52 | 7.47 | 8.75 | 10.36 | 12.17 | 13.48 |
| 50 | -0.38 | 0.24 | 6.06 | 6.55 | 7.49 | 8.78 | 10.39 | 12.22 | 13.52 |
| 55 | -0.38 | 0.24 | 6.08 | 6.57 | 7.52 | 8.81 | 10.43 | 12.26 | 13.58 |
| 60 | -0.38 | 0.24 | 6.11 | 6.60 | 7.56 | 8.85 | 10.48 | 12.32 | 13.64 |
| 65 | -0.38 | 0.24 | 6.14 | 6.64 | 7.60 | 8.90 | 10.54 | 12.39 | 13.71 |
| 70 | -0.38 | 0.24 | 6.18 | 6.68 | 7.64 | 8.95 | 10.60 | 12.46 | 13.79 |
| 75 | -0.38 | 0.24 | 6.22 | 6.72 | 7.69 | 9.01 | 10.67 | 12.54 | 13.88 |
| 80 | -0.38 | 0.24 | 6.26 | 6.76 | 7.74 | 9.07 | 10.74 | 12.62 | 13.97 |
| 85 | -0.38 | 0.24 | 6.30 | 6.81 | 7.79 | 9.13 | 10.81 | 12.71 | 14.07 |

LMS: lambda, mu, and sigma; SPPB: Short Physical Performance Battery. Degree of freedom (L = 1, M = 2.1, and S = 1).

**
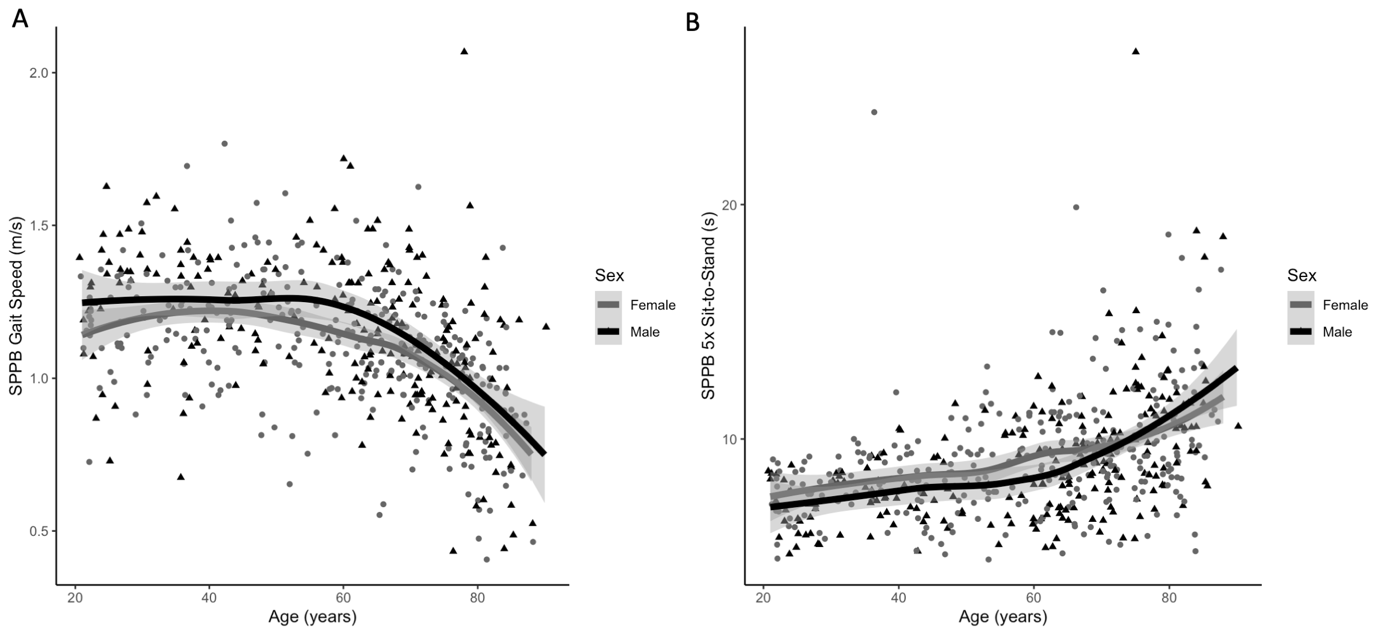
**

**Supplementary Figure S1.** Smooth local regression and 95% confidence intervals for individual SPPB subtests: 8-ft gait speed (A) and 5-times repeated chair sit-to-stand time (B) in males (black line, triangle) and females (grey line, circle).
